# Unraveling UVA1-Induced Photomodifications of Eumelanin and Pheomelanin in Human Skin: Insights into Pigment Darkening

**DOI:** 10.3390/ijms27093973

**Published:** 2026-04-29

**Authors:** Shosuke Ito, Juliette Sok, Yukiko Nakanishi, Kazumasa Wakamatsu, Sandra Del Bino

**Affiliations:** 1 Institute for Melanin Chemistry, Fujita Health University, Toyoake 470-1192, Japan; 2 L’Oreal Research and Innovation, 93601 Aulnay-sous-Bois, France

**Keywords:** melanins, eumelanin, pheomelanin, UVA1, pigment darkening, photooxidation, photodegradation

## Abstract

UVA exposure elicits immediate and persistent pigment darkening of the skin, which is thought to result from the oxidation and polymerization of existing melanin and/or precursors. Melanocytes produce eumelanin and pheomelanin. Eumelanin consists of 5,6-dihydroxyindole (DHI) and 5,6-dihydroxyindole-2-carboxylic acid (DHICA), while pheomelanin consists of benzothiazine and benzothiazole units. Melanins can be analyzed by quantifying specific degradation products using HPLC. Specifically, eumelanin can be analyzed as pyrrole-2,3,5-tricarboxylic acid (PTCA) and pyrrole-2,3-dicarboxylic acid (PDCA), specific degradation products of DHICA and DHI, respectively. Benzothiazole pheomelanin can be analyzed as thiazole-2,4,5-tricarboxylic acid (TTCA), whereas benzothiazine pheomelanin is analyzed as 4-amino-3-hydroxyphenylalanine (4-AHP) and 3-amino-4-hydroxyphenylalanine (3-AHP). Upon UVA exposure, melanins undergo structural modifications. Eumelanin undergoes oxidative cleavage to free pyrrole-2,3,5-tricarboxylic acid (Free PTCA) and undergoes cross-linking to form pyrrole-2,3,4,5-tetracarboxylic acid (PTeCA). UVA exposure of pheomelanin induces oxidative conversion from the benzothiazine to the benzothiazole. Nevertheless, these structural modifications have never been previously characterized in human skin. In this study, we exposed ex vivo skin to increasing UVA1 doses (60, 90 and 120 J/cm^2^; *n* = 6 in triplicate) and characterized the induced pigment darkening before, immediately, and 2 h after exposure through colorimetry, HPLC and spectrophotometry. The results showed changes in the CIELAB colorimetric parameters, namely a decrease in Luminance L*, the yellow-blue component b* and the Individual Typology Angle (ITA) in UVA1-exposed samples, indicative of skin darkening. In parallel, UVA1 exposure induced significant modifications of the levels of absorbance at 500 nm (A500) and melanin markers PTCA, PTeCA, PDCA, TTCA, and 4-AHP, as well as in the ratios of various markers, such as PTeCA/PTCA, Free/Total PTCA, and TTCA/4-AHP, indicative of photooxidation/degradation of melanins. Our study provides the first evidence of UVA1-induced modifications of melanins associated with pigment darkening occurring in human skin.

## 1. Introduction

UVA1 (340–400 nm) represents up to 80% of total UV reaching the Earth’s surface and contributes to skin photoaging [1,2], photocarcinogenesis [3,4,5,6], immune suppression [4,7,8] and pigment darkening [9,10]. The pigmentary response of human skin to sun exposure can be subdivided into three phases: Immediate Pigment Darkening (IPD), Persistent Pigment Darkening (PPD) and delayed tanning [11,12,13,14,15,16,17,18]. IPD is a blue-gray transitory darkening of the skin which appears during or just after UV exposure and fades within a few minutes to hours after exposure. When the skin is exposed to sufficient doses of UVA (>10 J/cm^2^ UVA 320–400 nm), IPD is more intense and is followed by PPD, a brownish pigmentation that peaks at 2 h after exposure, and can last 24 h or longer [19,20,21], eventually blending with delayed tanning, which takes several days to become apparent. While delayed tanning, especially after UVB exposure, has been well-characterized and requires new melanin synthesis [22,23,24], IPD and PPD are much less understood. These responses are induced by UVA (320–400 nm) exposure and are thought to involve redistribution of melanosomes—the melanin-containing organelles—within keratinocytes, as well as chemical modifications of preexisting melanins and/or melanogenic precursors [22,25,26]. Nevertheless, the UVA1 (340–400 nm)-induced changes in melanins underlying the clinical manifestations of IPD and PPD in human skin have never been fully analyzed.

Melanocytes synthesize two types of melanins: the brown-black eumelanin and the yellow orange pheomelanin [27]. Eumelanin consists of 5,6-dihydroxyindole (DHI) and 5,6-dihydroxyindole-2-carboxylic acid (DHICA) moieties, while pheomelanin consists of benzothiazine and benzothiazole moieties. These melanin monomers can be quantified by high-performance liquid chromatography (HPLC) through specific degradation products. After Alkaline Hydrogen Peroxide Oxidation (AHPO), pyrrole-2,3,5-tricarboxylic acid (PTCA) can be analyzed as a specific degradation product of DHICA-eumelanin, pyrrole-2,3-dicarboxylic acid (PDCA) as a specific degradation product of DHI-eumelanin, and thiazole-2,4,5-tricarboxylic acid (TTCA) as a specific degradation product of benzothiazole-pheomelanin [28,29]. Benzothiazine-pheomelanin content can be assessed after hydrolysis with hydroiodic acid (HI) through the measurement of the specific markers 4-amino-3-hydroxyphenylalanine (4-AHP) and 3-amino-4-hydroxyphenylalanine (3-AHP) [28,30]. Furthermore, total melanin content can be analyzed by spectrophotometry after solubilization with Soluene-350 and the measurement of the absorbance at 500 nm [31]. Recently, we analyzed the melanin content in skin samples with variable constitutive pigmentation [32,33]. Our results confirmed an increase in the total melanin content with constitutive pigmentation and showed an increase in eumelanin and benzothiazole-pheomelanin but not benzothiazine-pheomelanin, with constitutive pigmentation. The results also revealed a constant eumelanin/pheomelanin ratio regardless of the degree of pigmentation and interestingly showed that the human epidermis comprises approximately 41% DHICA-eumelanin, 35% DHI-eumelanin, 20% benzothiazole-pheomelanin, and 4% benzothiazine-pheomelanin, regardless of the degree of pigmentation [33].

IPD results from UVA-induced modifications of preexisting melanins, namely the oxidative conversion of the 5,6-dihydroxyindole form of DHI and DHICA to the 5,6-indolequinone form. This process leads to an overall increase in visible light absorption and a decrease in UVA absorption, resulting in the darkening of preexisting eumelanin [14,25,33,34]. The IPD may subsequently fade away through the reduction of indolequinone back to its original dihydroxyindole form. However, following higher UVA doses, irreversible chemical modification of melanin occurs, inducing PPD.

**Figure 1:**
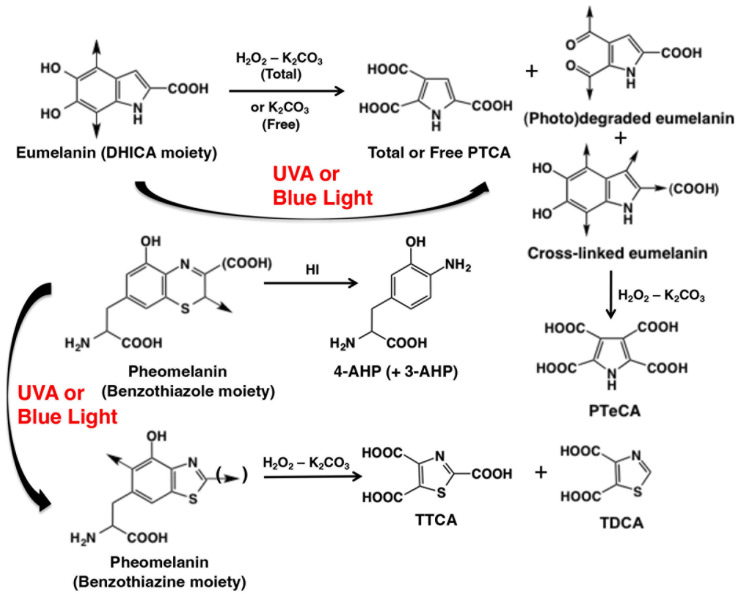
Photo-induced structural modifications of eumelanin (EM) and pheomelanin (PM) and their molecular markers. UVA or short-wavelength visible light induced structural modifications of EM and PM. For the sake of simplicity, only the 5,6-dihydroxyindole-2-carboxylic acid (DHICA) moiety is depicted, and the 5,6-dihydroxyindole (DHI) moiety is not depicted. Photo-induced modifications of EM give free pyrrole-2,3,5-tricarboxylic acid (PTCA) and pyrrole-2,3,4,5-tetracarboxylic acid (PTeCA) through photooxidation (the latter through cross-linking at C3 of the pyrrole ring of DHICA). Alkaline hydrogen peroxide oxidation (AHPO) of EM gives PTCA and PDCA from the DHICA and DHI moieties, respectively. The benzothiazine moiety in PM gives 4-amino-3-hydroxyphenylalanine (4-AHP; and 3-AHP) upon hydroiodic acid (HI) hydrolysis. UVA or visible light induces conversion of the benzothiazine to the benzothiazole unit. The latter gives thiazole-2,4,5-tricarboxylic acid (TTCA) and thiazole-4,5-dicarboxylic acid (TDCA) upon AHPO. Reproduced with permission from S. Ito [34].

The literature data on natural or synthetic melanins, namely human black hair (mostly eumelanin), synthetic DHICA-melanin, or cultured melanocytes exposed to quite high UVA doses, showed that these conditions induced the degradation/modification of both eumelanins and pheomelanins that can be evaluated by HPLC [34,35,36] (Figure 1). Upon UVA exposure, the indolequinone moieties of DHICA and DHI moieties undergo oxidative cleavage, generating Free PTCA and thereby increasing both the Free PTCA level and the Free/Total PTCA ratio [34]. In parallel, eumelanin photodegradation induces a decrease in Total PTCA and Total PTCA/A500 ratio. The PTCA levels obtained by AHPO and K_2_CO_3_ extraction under reducing conditions were referred to as “Total PTCA” and “Free PTCA”, respectively. UVA also induces cross-linking of the indolequinone moieties of DHICA and DHI, generating PTeCA upon AHPO and consequently increasing PTeCA levels and the PTeCA/PTCA ratio. For pheomelanin, UVA exposure induces the oxidative conversion of benzothiazine-pheomelanin to benzothiazole-pheomelanin. This leads to an increase in TTCA alongside a decrease in 4-AHP and 3-AHP, resulting in a decrease in 4-AHP/3-AHP ratio and an increase in the TTCA/4-AHP ratio [34].

To gain better insights into human skin darkening, our objective was to characterize the UVA1-induced changes in melanins in vitro. We exposed ex vivo skin to UVA1 (60, 90, and 120 J/cm^2^) and analyzed the pigmentary response at different time points—before, immediately after (designated as 0 h) and 2 h post-exposure—using colorimetry, HPLC, and spectrophotometry.

## 2. Results

### 2.1. Macroscopic Aspect of Ex Vivo Skin and Colorimetric Parameters

Eighteen ex vivo skin specimens (Tan = 2, Brown = 11, and Dark = 5) were included in the study based on their Individual Typology Angle (ITA) values [37]. These specimens were exposed to three increasing doses of UVA1 (60, 90, and 120 J/cm^2^), with six studies per dose tested in triplicates per condition. The results were averaged to compensate for/mitigate the inter-individual variability in human skin (Table S1).

**Figure 2:**
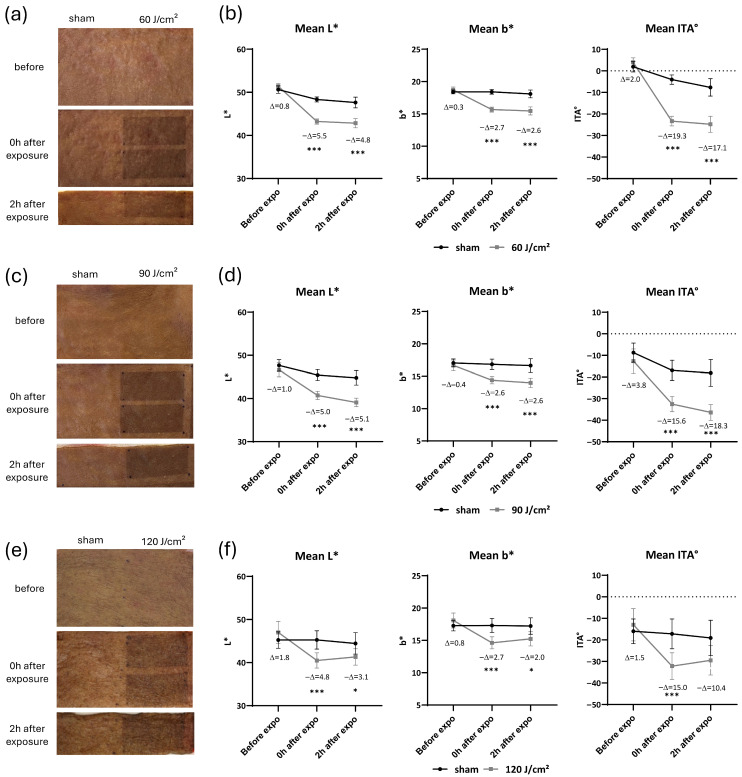
Macroscopic aspect of ex vivo skin and colorimetric parameters after exposure to 60, 90, and 120 J/cm^2^ UVA1. (**a**,**c**,**e**) Photographs of ex vivo skin at sham or exposed site before, immediately, and 2 h after exposure at 60, 90, and 120 J/cm^2^ UVA1. (**b**,**d**,**f**) Changes in L*, b* parameters and Individual Typology Angle (ITA) before, immediately, and 2 h after exposure at 60, 90, and 120 J/cm^2^ UVA1. Data were averaged from six independent experiments with standard error of the mean (SEM). Student’s *t*-test for paired samples (two-tailed), * = *p* < 0.05, *** = *p* < 0.001.

Ex vivo skin samples were exposed to a UVA1 dose of 60 J/cm^2^, which induced a visible skin darkening both immediately and 2 h post-exposure. These changes were quantitatively confirmed via spectrocolorimeter (Figure 2a,b). Compared to unexposed sites, UVA1 exposure caused a significant decrease (*p* < 0.001) in the L* parameter (lightness) with ΔL* values of 5.5 and 4.8 at the immediate and 2 h marks, respectively. Similarly, a significant decrease (*p* < 0.001) in the b* parameter was observed (Δb* of 2.7 and 2.6), consistent with a more pronounced blue-gray skin appearance. Furthermore, ITA significantly decreased (*p* < 0.001), with ΔITA values of 19.3 and 17.1, further indicating skin darkening.

Increasing the UVA1 dose to 90 J/cm^2^ resulted in similar visible and measurable darkening. Significant colorimetric changes (*p* < 0.001) were observed: ΔL* of 5.0 and 5.1, Δb* of 2.6 and 2.6, and ΔITA of 15.6 and 18.3 (immediate and 2 h, respectively; Figure 2c,d). These shifts were comparable to those observed with 60 J/cm^2^ exposure.

A higher UVA1 exposure, namely 120 J/cm^2^, was then performed. This dose induced a visible and measurable skin darkening immediately and 2 h after the exposure with ΔL* of 4.8 and 3.1, Δb* of 2.7 and 2.0, ΔITA of 15.0 and 10.4 immediately and 2 h after exposure, respectively (Figure 2e,f). The magnitude of these colorimetric changes remained compared to the results of the two lower UVA1 doses.

### 2.2. Histology and Melanin Index

**Figure 3:**
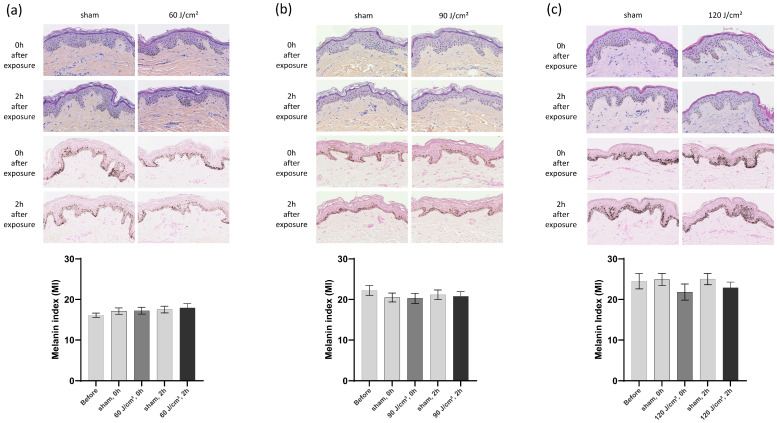
Histology (Hematoxylin–Eosin–Saffron, HES staining, first two rows of images within each panel (**a**–**c**)), melanin staining (Fontana–Masson, third and fourth rows of images within each panel (**a**–**c**)) and melanin index (MI) corresponding to the surface covered by the melanin staining, including the stratum corneum over the surface of the epidermis, were assessed before, immediately, and 2 h after exposure to 60 J/cm^2^ (**a**), 90 J/cm^2^ (**b**), and 120 J/cm^2^ (**c**). For the MI, data were averaged from six independent experiments with SEM. Student’s *t*-test for paired samples (two-tailed).

At a UVA1 dose of 60 J/cm^2^, histological Hematoxylin–Eosin–Saffron (HES) staining revealed no visible morphological cytotoxicity following exposure (Figure 3a). Fontana–Masson staining and melanin index (MI) measurements showed no significant changes in the melanin content. Similarly, at 90 J/cm^2^, HES staining showed no cytotoxicity, and no significant modifications in the melanin levels were observed (Figure 3b).

Even at the highest dose of 120 J/cm^2^, no cytotoxicity was detected; however, while Fontana–Masson staining and MI generally showed no significant modifications, some slight decreases in the melanin amount were noted (Figure 3c).

### 2.3. Melanin Marker Changes After Exposure to 60 J/cm^2^ UVA1

**Figure 4:**
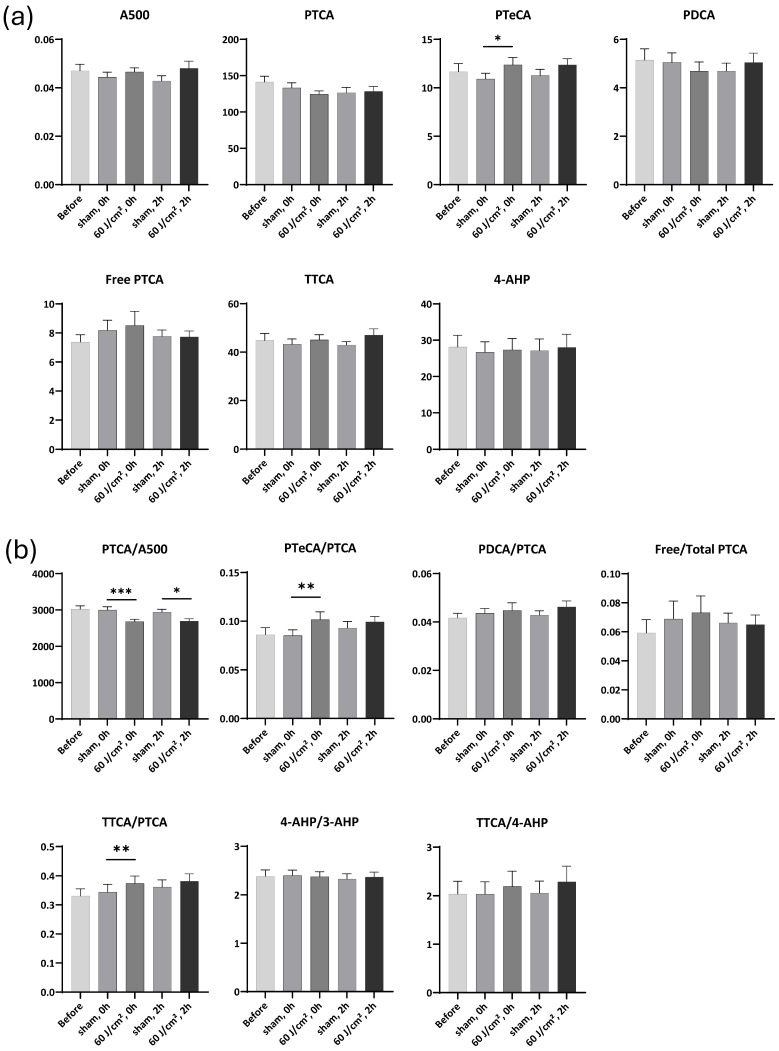
Melanin markers and ratios after exposure to 60 J/cm^2^ UVA1. (**a**) Changes in melanin markers before, immediately, and 2 h after exposure. (**b**) Changes in marker ratios before, immediately, and 2 h after exposure. Data were averaged from six independent experiments with SEM. Student’s *t*-test for paired samples (one-tailed), * = *p* < 0.05, ** = *p* < 0.01, *** = *p* < 0.001.

For the melanin markers, no statistically significant changes were observed in A500, PTCA, PDCA and Free PTCA following the UVA1 exposure at a dose of 60 J/cm^2^, although an upward trend was noted at 2 h (Figure 4a). In contrast, the exposure induced a significant 1.13-fold change (FC) increase in PTeCA immediately post-exposure (*p* < 0.05). Here, FC is defined as the ratio of marker levels in UVA1-exposed samples relative to sham-treated controls.

The UVA1 exposure led to a significant decrease in the PTCA/A500 ratio, immediately (0.89 FC, *p* < 0.001) and 2 h after exposure (0.92 FC, *p* < 0.05), suggesting eumelanin photodegradation. It also induced a significant 1.19 FC increase in the PTeCA/PTCA ratio immediately after exposure (*p* < 0.01), indicative of eumelanin cross-linking (Figure 4b). Conversely, the 60 J/cm^2^ UVA1 exposure induced no significant changes on PDCA/PTCA ratio.

Unlike its effects on eumelanin, the UVA1 exposure had minimal impact on the pheomelanin markers 4-AHP and TTCA (Figure 4a), or on the 4-AHP/3-AHP and TTCA/4-AHP ratios (Figure 4b). However, the UVA1 exposure induced a significant 1.09 FC increase (*p* < 0.01) in the TTCA/PTCA ratio immediately after exposure, reflecting a more rapid decline in PTCA. These results confirmed that eumelanin was more susceptible to impairment by the 60 J/cm^2^ UVA1 exposure than pheomelanin, which prompted us to increase the UVA1 dose in subsequent experiments.

### 2.4. Melanin Marker Changes After Exposure to 90 J/cm^2^ UVA1

**Figure 5:**
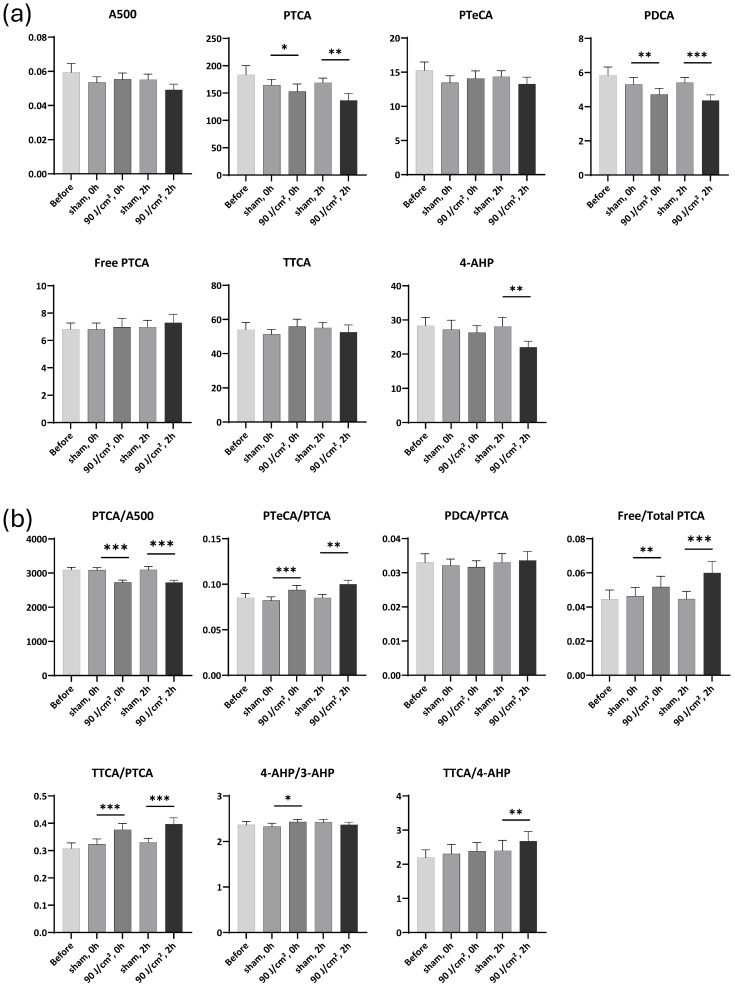
Melanin markers and ratios after 90 J/cm^2^ UVA1 exposure. (**a**) Changes in melanin markers before, immediately, and 2 h after exposure. (**b**) Changes in marker ratios before, immediately, and 2 h after exposure. Data were averaged from six independent experiments with SEM. Student’s *t*-test for paired samples (one-tailed), * = *p* < 0.05, ** = *p* < 0.01, *** = *p* < 0.001.

Regarding the melanin markers, UVA1 exposure at a dose of 90 J/cm^2^ induced no statistically significant changes in A500, PTeCA and Free PTCA. However, it caused significant 0.93- and 0.81 FC decreases in PTCA (*p* < 0.05 and *p* < 0.01, respectively), and 0.89- and 0.81 FC decreases in PDCA (*p* < 0.01 and *p* < 0.001, respectively) (Figure 5a). These results indicate that both DHICA and DHI units in eumelanin were photodegraded.

Exposure to 90 J/cm^2^ induced a significant 0.88 FC (*p* < 0.001) decrease in the PTCA/A500 ratio immediately and 2 h after exposure, respectively, indicative of eumelanin photodegradation (Figure 5b). It also induced a significant 1.19 FC (*p* < 0.01 and *p* < 0.001, respectively) increase in the Free/Total PTCA ratio 2 h after exposure, further suggesting eumelanin photodegradation. Furthermore, the UVA1 exposure induced a significant increase in the PTeCA/PTCA ratio immediately and 2 h after exposure (1.14 and 1.18 FC; *p* < 0.001 and *p* < 0.01, respectively), which is indicative of eumelanin cross-linking. UVA1 exposure induced no significant changes on PDCA/PTCA ratio.

As regards pheomelanin, the 90 J/cm^2^ UVA1 exposure induced no statistically significant changes in TTCA, but it did cause a significant 0.78 FC (*p* < 0.01) decrease in 4-AHP 2 h after exposure (Figure 5a). The UVA1 exposure induced a significant 1.12 FC (*p* < 0.01) increase in the TTCA/4-AHP ratio 2 h after exposure, indicative of pheomelanin photooxidation (Figure 5b). It also induced significant 1.17 and 1.20 FC (*p* < 0.001) increases in the TTCA/PTCA ratio immediately and 2 h after exposure, respectively.

### 2.5. Melanin Marker Changes After Exposure to 120 J/cm^2^ UVA1

**Figure 6:**
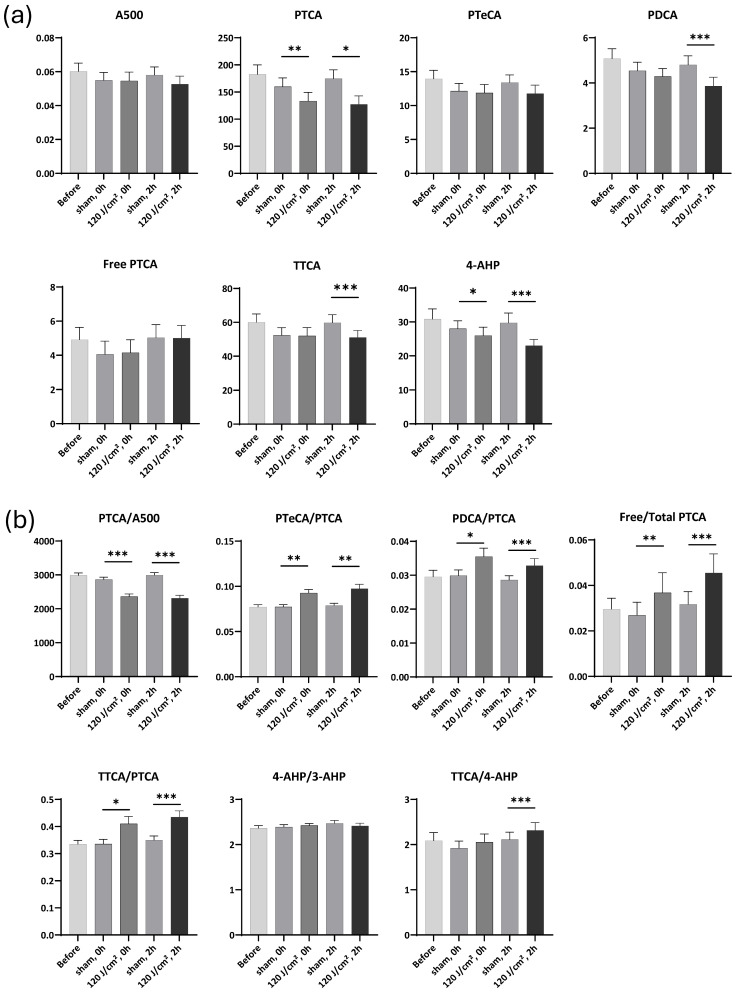
Melanin markers and ratios induced by 120 J/cm^2^ UVA1. (**a**) Changes in melanin markers before, immediately, and 2 h after exposure. (**b**) Changes in marker ratios before, immediately, and 2 h after exposure. Data were averaged from six independent experiments with SEM. Student’s *t*-test for paired samples (one-tailed), * = *p* < 0.05, ** = *p* < 0.01, *** = *p* < 0.001.

Again, for the melanin markers, UVA1 exposure at a dose of 120 J/cm^2^ induced no statistically significant changes in A500, PTeCA, and Free PTCA. However, it caused significant 0.83 and 0.73 FC decreases in PTCA (*p* < 0.01 and *p* < 0.05, respectively), and a 0.80 FC decrease in PDCA (*p* < 0.001) 2 h after exposure (Figure 6a). These decreases were similar to those observed at 90 J/cm^2^ but were more pronounced.

The 120 J/cm^2^ UVA1 exposure induced significant 0.82 and 0.77 FC (*p* < 0.001 for both) decreases in the PTCA/A500 ratio immediately and 2 h after exposure. Significant 1.23 and 1.32 FC (*p* < 0.01 and *p* < 0.001, respectively) increases in the Free/Total PTCA were also observed, both indicative of the photodegradation of eumelanin (Figure 6b). In parallel, the 120 J/cm^2^ exposure induced significant 1.20 and 1.23 FC (*p* < 0.01 for both) increases in the PTeCA/PTCA ratio, indicative of eumelanin cross-linking. Furthermore, the exposure induced 1.19 and 1.15 FC (*p* < 0.05 and *p* < 0.001, respectively) increases in the PDCA/PTCA ratio, showing that PDCA decreased less than PTCA values. This indicated that DHI units were less susceptible to UVA1-induced oxidative degradation than DHICA units.

For pheomelanin, the results showed a significant 0.86 FC (*p* < 0.001) decrease in TTCA 2 h after exposure, indicative of pheomelanin photodegradation. Significant 0.93 and 0.77 decreases (*p* < 0.05 and *p* < 0.001, respectively) in 4-AHP were also observed (Figure 6a), resulting in a significant 1.10 FC increase in the TTCA/4-AHP ratio. This is indicative of the conversion of benzothiazine to benzothiazole units 2 h after exposure (*p* < 0.001) (Figure 6b). Furthermore, the UVA1 exposure induced significant 1.22 and 1.24 FC (*p* < 0.05 and *p* < 0.001, respectively) increases in the TTCA/PTCA ratio immediately and 2 h after the exposure, indicating a faster degradation of eumelanin compared to pheomelanin.

Altogether, the results showed that exposure of ex vivo dark and tan human skin samples to a single UVA1 dose (60, 90 and 120 J/cm^2^) induced changes in the CIELAB colorimetric parameters—namely, decreases in L* and b*, and ITA—all indicative of skin darkening. These colorimetric changes were accompanied by dose-dependent modifications of various markers and their ratios: specifically, decreases in PTCA, 4-AHP, and TTCA, and increases in PTeCA/PTCA, Free/Total PTCA, and TTCA/4-AHP ratios. These findings are indicative of photodegradation and photooxidation (including cross-linking) of melanins. These results also demonstrated a dose-dependent greater impairment of eumelanin compared to pheomelanin and, within eumelanin, a greater impairment of DHICA units compared to DHI units at the highest UVA1 dose.

### 2.6. Dose-Dependent Changes in Melanin Markers

**Figure 7:**
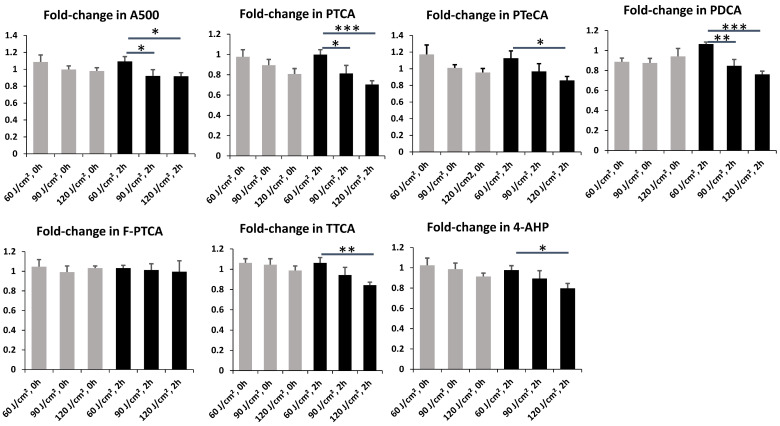
Fold changes (FCs) in melanin markers depending on UVA1 doses of 60, 90, and 120 J/cm^2^. Data were averaged from six independent experiments with SEM. Student’s *t*-test for unpaired samples (one-tailed), * = *p* < 0.05, ** = *p* < 0.01, *** = *p* < 0.001.

UVA1 exposure at 60 J/cm^2^ tended to induce photooxidation, as indicated by the increased melanin markers. In contrast, exposures at 90 and 120 J/cm^2^ appeared to trigger photodegradation, evidenced by a decrease in these markers. Therefore, we compared FCs immediately and 2 h post-exposure at 60, 90, and 120 J/cm^2^ to evaluate dose dependency (Figure 7).

Dose-dependent decreases were observed in A500, PTCA, PTeCA, PDCA, TTCA, and 4-AHP at 2 h post-irradiation, with statistical significance between the 60 and 120 J/cm^2^ doses. Similar but less pronounced decreases were also observed immediately after exposure, though these did not reach statistical significance. Notably, most markers exhibited an FC greater than 1.00 at 60 J/cm^2^, which progressively declined to below 1.00 at 90 and 120 J/cm^2^. These results suggested that at the lower dose of 60 J/cm^2^, the primary effect of UVA1 exposure is photooxidation, which is superseded by photodegradation at higher doses (90 and 120 J/cm^2^).

### 2.7. Correlation of A500 with Melanin Markers

**Figure 8:**
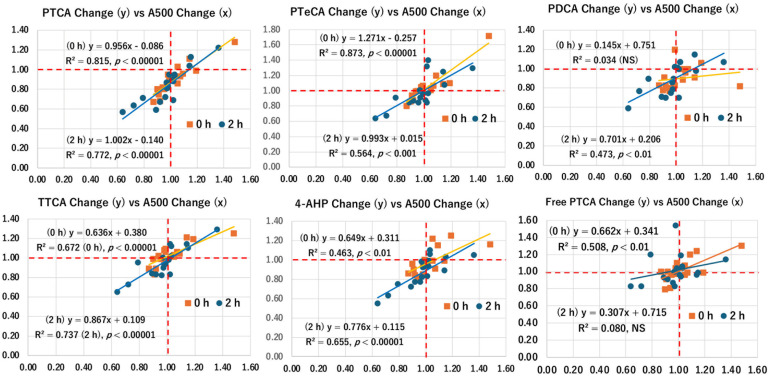
Correlation between A500 changes vs. melanin marker changes. Correlation graphs were prepared for FCs immediately after exposure (0 h) and 2 h after exposure (2 h). Dashed red lines indicate FC = 1.00.

In this study, A500 and marker values tended to increase at 60 J/cm^2^ (FC > 1.00), followed by a decrease at 90 and 120 J/cm^2^. Therefore, we evaluated the correlation between the FC of A500 and those of melanin markers. Correlation graphs were prepared for the FC of immediately (0 h) and 2 h after UVA1 exposure (Figure 8). PTCA showed an excellent correlation both immediately and 2 h after exposure (R^2^ = 0.815 and 0.772, respectively). Furthermore, the slopes of the correlation graph were almost identical (0.96 vs. 1.00), suggesting that structural changes in melanins proceeded similarly during the 2 h period. In addition, PTeCA, PDCA (2 h), Free PTCA (0 h), TTCA, and 4-AHP also showed good to excellent correlations with similar slopes.

FC less than 1.00 for A500 should be ascribed to photodegradation. In contrast, FC values greater than 1.00 for A500 are due to the darkening of melanin resulting from the oxidation of dihydroxyindole to the indolequinone form [25]. But what would cause the increase in PTCA? We speculate that the oxidized indolequinone of the DHICA moiety is more easily cleaved by AHPO than the reduced dihydroxyindole form, leading to higher yields of PTCA. Likewise, oxidation to the indolequinone in DHI moiety leads to a corresponding increase in PDCA. Increased yields of PTeCA (FC > 1.00) after UVA1 oxidation are attributed to cross-linking occurring after photooxidation to the DHICA-quinone moiety (see Figure 1). Increased yields of TTCA can be ascribed to the photooxidative conversion of the benzothiazine to the benzothiazole moiety [36]. Finally, increased yields of 4-AHP could be due to the photochemical production of pheomelanin from its precursor 5-*S*-cysteinyldopa [38].

### 2.8. Light Skin Response

**Figure 9:**
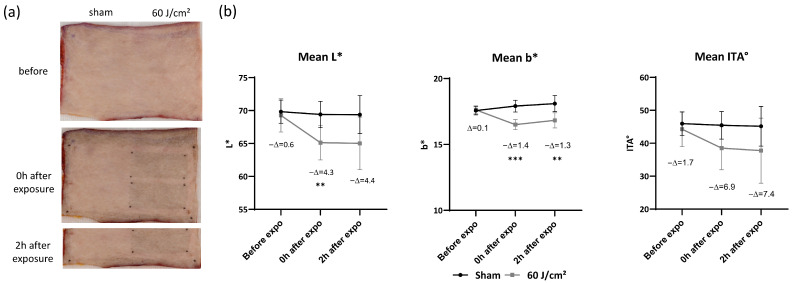
Macroscopic aspect of light ex vivo skin and colorimetric parameters after exposure to 60 J/cm^2^ UVA1. (**a**) Photographs of ex vivo skin at sham or exposed site before, immediately, and 2 h after exposure. (**b**) Changes in L* and b* parameters and Individual Typology Angle (ITA) before, immediately, and 2 h after exposure. Data were averaged from five independent experiments with SEM. Student’s *t*-test for paired samples (two-tailed), ** = *p* < 0.01, *** = *p* < 0.001.

In order to achieve precise quantification of melanin-related biomarkers in poorly pigmented conditions, we investigated the impact of UVA1 on fair skin types. A total of fifteen specimens (3 Very Light; 12 Light) were exposed to increasing doses of UVA1 (60, 90, and 120 J/cm^2^). All exposure conditions induced visible skin darkening both immediately and 2 h post-exposure. Macroscopic aspect and colorimetric parameters after exposure to 60 J/cm^2^ are illustrated in Figure 9.

Compared to unexposed sites, 60 J/cm^2^ UVA1 exposure caused a significant decrease in the L* parameter, with a ΔL* value of 4.3 (*p* < 0.01) immediately and 4.4 (not significant) 2 h post-exposure, and a significant decrease in the b* parameter with Δb* of 1.4 and 1.3 (*p* < 0.001 and *p* < 0.01) immediately and 2 h after exposure (Figure 9). Furthermore, ITA decreased with ΔITA values of 6.9 and 7.4 (not significant) immediately and 2 h after exposure. Note that the ΔITA was smaller for lighter skin compared to darker skin.

As for dark skin types, HES staining revealed no cytotoxicity for the three UVA1 doses. Furthermore, Fontana–Masson staining and MI showed no significant modifications.

**Figure 10:**
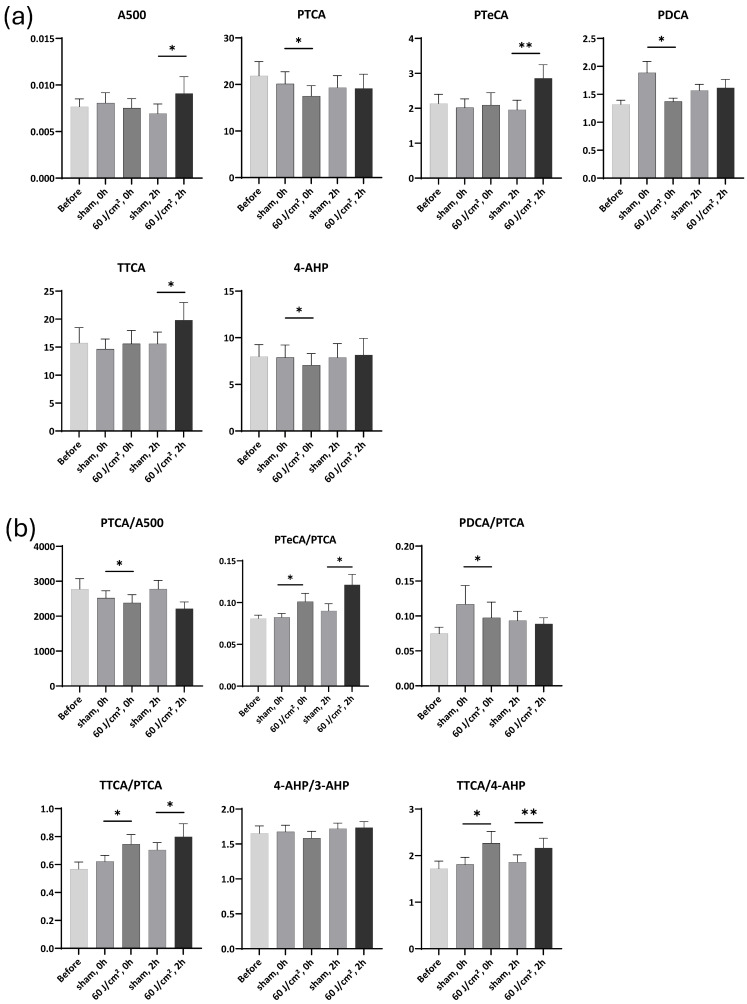
Melanin markers and ratios after 60 J/cm^2^ UVA1 exposure on light skin. (**a**) Changes in melanin markers before, immediately, and 2 h after exposure. (**b**) Changes in marker ratios before, immediately, and 2 h after exposure. Data were averaged from five independent experiments with SEM. Student’s *t*-test for paired samples (one-tailed), * = *p* < 0.05, ** = *p* < 0.01.

For the melanin markers and ratios, altogether the results demonstrate that the light skin response is similar to that of dark skin, but in contrast to the latter, pheomelanin is impacted earlier, as soon as 60 J/cm^2^ exposure (see Figures S2 and S3 and Table S1 for average data after 60, 90, and 120 J/cm^2^ UVA1). The response to 60 J/cm^2^ is detailed in Figure 10a,b. Exposure to 60 J/cm^2^ induced a statistically significant 1.28 FC increase in A500 (*p* < 0.05) 2 h after exposure, indicative of eumelanin photooxidation, and a 0.83 FC decrease in PTCA (*p* < 0.05) immediately after exposure, representative of eumelanin photodegradation (Figure 10a). Exposure to 60 J/cm^2^ also induced a 1.17 FC increase in PTeCA (*p* < 0.01) 2 h after exposure, indicative of eumelanin cross-linking, and a 0.75 FC decrease in PDCA (*p* < 0.05) immediately after exposure. Free PTCA could not be detected (as for the two other UVA1 doses).

The UVA1 exposure induced a significant 0.92 FC (*p* < 0.05) decrease in the PTCA/A500 ratio immediately after exposure (Figure 10b), indicative of eumelanin photodegradation. It also induced a significant 1.17 and 1.29 FC (*p* < 0.05 in both) increase in the PTeCA/PTCA ratio immediately and 2 h after exposure, indicative of eumelanin cross-linking. Furthermore, the exposure induced a 0.91 FC (*p* < 0.05) decrease in the PDCA/PTCA ratio immediately after exposure.

For pheomelanin, the results showed a significant 1.06 FC increase (*p* < 0.05) 2 h after exposure and a 0.81 (*p* < 0.05) decrease in 4-AHP immediately after the exposure (Figure 10a), resulting in a significant 1.13 and 1.04 FC increase in the TTCA/4-AHP ratio immediately and 2 h after (*p* < 0.05 in both), indicative of the conversion of benzothiazine to benzothiazole units (Figure 10b). Furthermore, the UVA1 exposure induced significant 1.21 and 1.17 FC (*p* < 0.05 in both) increases in the TTCA/PTCA ratio immediately and 2 h after the exposure. Overall results demonstrate that at 60 J/cm^2^, light skin (in addition to eumelanin photodegradation (decreased PTCA and PTCA/A500) and cross-linking (increased PTeCA and PTeCA/PTCA), also shown for dark skin at the same dose) undergoes pheomelanin photo-oxidation, as illustrated by decreased 4-AHP, increased TTCA and TTCA/4-AHP, which were not evidenced for dark skin.

Exposure to 90 J/cm^2^, illustrated in Figure S2 and Table S1, showed eumelanin photodegradation (decrease in PTCA and PTCA/A500), eumelanin cross-linking (increase in PTeCA and PTeCA/PTCA) and pheomelanin photo-oxidation (decrease in 4-AHP and 4/3-AHP; increase in TTCA/4-AHP) results quite similar to what was observed previously with dark skin. The 120 J/cm^2^ results (Figure S3 and Table S1) again showed eumelanin photooxidation (decreased PTCA), cross-linking (increased PTeCA/PTCA) and eumelanin photodegradation (decreased PTCA/A500 and increased Free/Total PTCA), along with pheomelanin photooxidation (decrease in 4-AHP and increased TTCA/4-AHP).

## 3. Discussion

Our study presents the first evidence of UVA1-induced modifications of melanins associated with pigment darkening occurring within human skin. This allowed us to thoroughly explore the structural changes in melanins—namely photooxidation, photodegradation, and cross-linking—which had previously been investigated only in other models, such as synthetic melanins, human hair, and cultured melanocytes exposed to high UVA doses [34,35,36].

Chemical analysis of melanin requires large sample volumes and destructive processes such as skin excision, epidermal separation, and freeze-drying. Such procedures could not, therefore, be ethically and practically performed on human volunteers, especially when repeated biopsies from multiple sites are required. Instead, we utilized ex vivo skin samples. Despite the inherent lack of systemic support, ex vivo human skin models are widely recognized in dermatological research as valuable proxies for simulating in vivo human skin responses. They preserve the native three-dimensional architecture, including diverse cell populations (keratinocytes, melanocytes, fibroblasts, resident immune cells such as Langerhans cells, lymphocytes, and macrophages), the dermal matrix, and the local antioxidant environment of the integument [39,40]. Ex vivo skin samples were exposed to realistic and achievable UVA1 doses. For relevance, the dose of 60 J/cm^2^ is equivalent to a 3 h afternoon exposure in São Paulo during summer, or an afternoon exposure in Paris during spring, representing a highly physiological dose. The 90 J/cm^2^ dose corresponds to midday to afternoon exposure in São Paulo during summer, or a full day in Paris during spring. The 120 J/cm^2^ dose corresponds to a full day exposure in São Paulo during summer [41,42]. Although 120 J/cm^2^ is a high dose, its inclusion was scientifically necessary to robustly demonstrate the dose-dependent nature of the observed biological effects. Furthermore, histological examination (HES staining) demonstrated no observable cytotoxicity even at the highest dose of 120 J/cm^2^, confirming the biosafety of these doses. Our selection is supported by previous clinical literature, where doses in the range of 30–60 J/cm^2^ have been safely used to observe skin darkening without adverse clinical effects [43].

Our results also demonstrate that in parallel with significant visual and colorimetric changes—accompanied by only a small change in melanin content, consistent with UVA literature [44]—there are small but very significant dose-dependent changes in melanin markers and ratios. These changes are indicative of photooxidation, photodegradation, and cross-linking of melanins. The fact that these changes are subtle can be interpreted by assuming that they require modifications throughout the entire mass of melanosome granules, whereas visual and colorimetric changes may primarily reflect alterations on the surface of melanosome granules. These results reinforce the hypothesis that both structural modifications of melanins and the redistribution of melanosomes in keratinocytes may contribute to the observed skin darkening [22,25,26].

**Figure 11:**
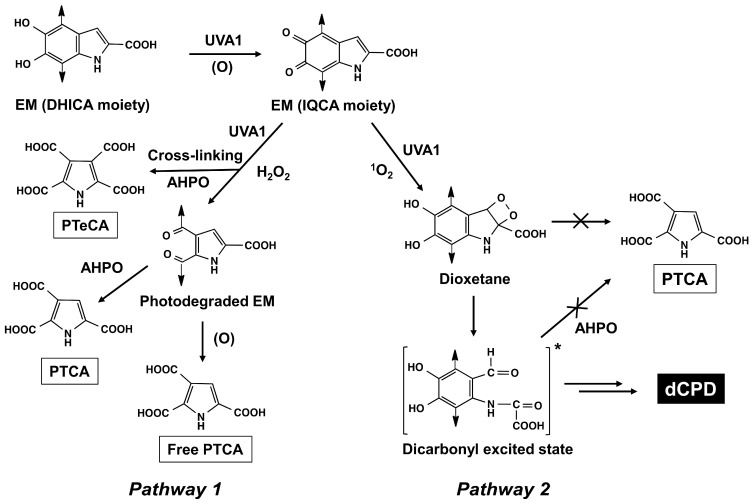
Possible pathways for UVA1-induced oxidation of DHICA-eumelanin (EM). For the sake of clarity, the DHI moiety is not shown. Photooxidation of the DHICA moiety yields an indolequinone-carboxylic acid (IQCA) moiety. The oxidative cleavage of the IQCA moiety proceeds via two pathways: Pathways 1 and 2. In Pathway 1, oxidative cleavage of the quinone ring occurs with hydrogen peroxide to produce photodegraded EM, yielding Total PTCA upon AHPO or liberating Free PTCA. The IQCA moiety undergoes cross-linking at C3 of the pyrrole ring, yielding PTeCA upon AHPO. In Pathway 2, singlet oxygen attacks the pyrrole ring, yielding a dicarbonyl excited state through a dioxetane intermediate. This pathway leads to the generation of delayed cyclobutene pyrimidine dimer (dCPD; [49,50]). Importantly, Pathway 2 does not yield PTCA upon AHPO. [ ]* indicates the excited state.

Two pathways are possible for the UVA1-induced oxidative degradation of eumelanin: Pathway 1 involves the production of PTCA upon AHPO, while Pathway 2 does not (Figure 11). Among melanin markers, PTCA (and PDCA) exhibited the most significant decrease when the UVA1 dose was increased to 120 J/cm^2^ (Figure 7). This decrease can be ascribed to the photodegradation of eumelanin by singlet oxygen generated during UVA1 exposure (Pathway 2). Ito et al. confirmed that not only the superoxide radical but also singlet oxygen are photogenerated upon UVA irradiation of synthetic DHICA-melanin [45]. In this regard, Mokrzyński et al. recently compared the reactivity of synthetic Dopa-melanin against UVA/Blue light (400 nm) and 0.1 M H_2_O_2_ and showed that while H_2_O_2_ induced cleavage of the catechol/quinone ring of DHICA, leading to an increase in Free PTCA and PTeCA through cross-linked eumelanin, singlet oxygen generated by UVA/Blue light induced cleavage of the pyrrole ring of DHICA, leading to a decrease in Total PTCA [46]. How DHICA (and DHI) units are photodegraded by singlet oxygen remains speculative. However, it is possible that the attack of singlet oxygen to the pyrrole ring produces a highly reactive dioxetane intermediate [47,48] which gives rise to an excited state dicarbonyl moiety. This, in turn, leads to the production of delayed cyclobutane pyrimidine dimer (dCPD) [49,50,51]. Singlet oxygen is highly reactive and adds to conjugated double bonds within a molecule to generate dioxetane—a strained, high-energy four-membered ring structure that stores significant energy in its C-C and O-O bonds. After the dioxetane ring is formed, molecular twisting caused by thermal energy leads to ring cleavage. During this fragmentation, the C-C and O-O bonds are cleaved, leaving two carbonyls (dicarbonyls), one of which becomes electronically excited. Crucially, one of the two carbonyls produced by this reaction is born directly in an excited triplet-state rather than the ground state; this phenomenon is known as “chemiexcitation”. These excited triplet melanins or excited carbonyls then transfer their energy to surrounding ground-state oxygen (triplet oxygen), resulting in the generation of further singlet oxygen [49,52,53,54,55]. As depicted in Figure 11, this Pathway 2 yields structural motifs that do not yield PTCA upon AHPO due to the destruction of the pyrrole ring. Conversely, the attachment of H_2_O_2_ (generated from superoxide radicals) to the quinone ring produces photodegraded eumelanin that yields PTCA upon AHPO (Pathway 1). The present study thus provides indirect evidence for the generation of dCPD upon UVA1 exposure.

Photomodification of pheomelanin is evaluated based on TTCA, 4-AHP, and the TTCA/4-AHP ratio. In previous studies, UVA exposure of synthetic pheomelanin [36,56], human red hair [36], isolated bovine retinal pigment epithelium melanosomes, and human melanocytes [57] induced the conversion of the benzothiazine to the benzothiazole moiety. This results in an increase in TTCA, a decrease in 4-AHP, and a subsequent increase in the TTCA/4-AHP ratio. In the present study, 4-AHP levels were found to decrease at higher doses of UVA1 (Figure 7). Conversely, TTCA also decreased with increasing UVA1 doses. This observation contradicts previous studies [56,57] and warrants further clarification of underlying mechanisms. However, it would be possible that the benzothiazole moiety in pheomelanin reacts with singlet oxygen, leading to the decrease in TTCA observed at 120 J/cm^2^ UVA. In this regard, Szewczyk et al. demonstrated that synthetic pheomelanins not only generate singlet oxygen during UVA-to-visible light irradiation but also quench singlet oxygen generated from rose Bengal [58]. Indeed, it is known that thiazoles react with singlet oxygen through [4 + 2] cycloaddition reaction [59].

This study primarily relies on chemical degradation products (e.g., PTCA, TTCA, and 4-AHP) to infer structural changes but does not assess whether these photochemical modifications affect the antioxidant capacity, free radical scavenging ability, or photoprotective function of melanin. In this regard, we emphasize that the decrease in absorbance at 500 nm (A500) shown in this study (Figure 7 and Figure 8) results in a decrease in the photoprotective function (visible light absorption capacity) of melanin. In addition, photochemical modification of melanin should lead to a decrease in antioxidant capacity. A recent study showed that oxidative degradation of Dopa-melanin decreased its antioxidant properties: photodegraded melanin scavenged the 2,2-diphenyl-1-picrylhydrazyl (DPPH) radical and quenched singlet oxygen less effectively in parallel to the degree of photodegradation [46]. On the other hand, production of the singlet oxygen and superoxide radical increased significantly during photodegradation.

In contrast to a clinical study involving volunteers of diverse pigmentation and geographical origins (Europe, India and Africa) exposed to 30, 45–50, and 60 J/cm^2^ UVA1—which showed skin darkening associated with increasing ΔL* and Δb* with increasing doses [10]—we did not observe dose-dependent visual pigmentation or significant changes in colorimetric parameters between 60, 90, and 120 J/cm^2^. One possible explanation is that a plateau in visible pigmentation is reached at a dose of 60 J/cm^2^. Therefore, investigating lower UVA1 doses in our model would be of interest in the future. When comparing the differences in the colorimetric parameters before and after UVA1 exposure between dark and light skin, we observed the most important differences on ΔITA in dark skin, confirming that IPD/PPD are more pronounced in populations with darker skin type [60].

This study sheds light on the chemical origin of the difference between IPD and PPD that occurs immediately and a few hours after UVA exposure. Exposure to 60 J/cm^2^ tends to result in FC > 1.00, indicative of photooxidation. This may be related to IPD [25]. At 2 h after exposure to 90 and 120 J/cm^2^ UVA1, A500 and most melanin markers showed signs of progressive photodegradation (FC < 1.00). It should be emphasized that this photodegradation accompanies increases in visual pigmentation and the corresponding colorimetric changes. This apparent discrepancy can be explained by assuming that visual pigmentation, as seen in PPD, reflects changes on the surface of melanosomes, while changes in A500 and melanin markers reflect changes in the entire melanosome.

Interestingly, our results shed light on the varying susceptibilities of melanin pigments to UVA1 exposure. Specifically, at least in dark skin, pheomelanin appeared to be less affected by UVA1 than eumelanin. This observation is consistent with the “casing model” of mixed melanogenesis, in which a preformed pheomelanin core is encapsulated by a eumelanin surface [61,62]. We demonstrated previously that all skin color types contain a rather constant eumelanin/pheomelanin ratio with 74% of eumelanin and 26% of pheomelanin regardless of the degree of pigmentation [32,33]. In light skin, pheomelanin was shown to already be impacted after 60 J/cm^2^ UVA1. This might be due to a less important eumelanin surface, protecting the pheomelanin cores less effectively. Furthermore, at least in dark skin at the highest dose, DHI units appeared to be less impacted than DHICA units.

Although the observed structural modifications of melanin after UVA1 exposure are not drastic, they may still be harmful. Unlike tanning resulting from neo-melanogenesis, IPD and PPD are not photoprotective [23,63]. Oxidative degradation of eumelanin reduces its antioxidative properties [34,57], and the cross-linking of eumelanin (PTeCA) leads to a more crowded 3D structure. This reduces its capacity to sequester toxic ions such as Fe and Cu and to scavenge ROS [34,57]. Furthermore, the photomodification of pheomelanin involves the conversion of the benzothiazine moiety (4-AHP) to the benzothiazole moiety (TTCA), resulting in increased pheomelanin phototoxicity [56,64].

The clinical implications of this study are discussed herein. Melanin plays a crucial role in photoprotection, pigmentary disorders, and photoaging. This study primarily focuses on skin of color, defined by an Individual Typology Angle (ITA) < 28°, encompassing the Tan, Brown, and Dark skin types investigated. As reported by Krutmann et al. [65], individuals with skin of color face a significant risk of photodamage, including photoaging and photodermatoses, and exhibit a predisposition to pigmentary disorders. Despite these risks, adherence to photoprotective measures, such as sunscreen use, may be lower among individuals with skin of color.

This study highlights the critical importance of preventing UVA1-induced structural modifications of melanin, as these changes carry profound implications for photoaging and pigmentary disorders. Our findings suggest that the qualitative deterioration of skin melanin may mirror the age-dependent photodegradation observed in retinal pigment epithelium (RPE) melanin, a process known to contribute to various degenerative pathologies [66]. This parallel implies that UVA1-induced structural compromise is not merely a transient pigmentary change but a potential driver of long-term skin aging, particularly in individuals with skin of color who are predisposed to specific pigmentary complications.

A central mechanism identified here is the dual role of singlet oxygen as a mediator of both melanin degradation and delayed genotoxicity. Singlet oxygen facilitates the oxidative cleavage of the melanin polymer (Pathway 2) while simultaneously initiating “photochemistry in the dark” [46,47,48,49,50,51,55]. This process involves the formation of high-energy dioxetane intermediates and subsequent chemiexcitation, leading to the generation of delayed cyclobutane pyrimidine dimers (dCPD) that continues long after UVA1 exposure has ceased [47,48,49,50,51,52,53,54,55]. Such findings challenge the classical view of eumelanin as a purely photoprotective agent, demonstrating instead that preexisting pigment load can be a critical determinant of UV-induced genotoxicity. Recent evidence further suggests that intracellular melanin content, independent of ongoing melanogenesis, is sufficient to drive dCPD formation, potentially due to the translocation of oxidized melanin fragments into the nucleus [51].

While eumelanin possesses established antioxidant properties that correlate with reduced skin cancer incidence in heavily pigmented individuals [67,68], its capacity to mediate prolonged genotoxic stress reveals a complex, dualistic role in skin health [49,51,68]. Since the IPD and PPD observed in our study do not provide functional photoprotection, these results underscore a clear clinical imperative [23,63]. Preserving the chemical integrity of preexisting melanin is essential to mitigate delayed genomic stress and maintain the inherent antioxidant benefits of skin pigmentation [46,49,50,67,68].

Looking forward, the impact of UVA1 and other parts of the solar spectrum—most notably high-energy visible (HEV) light (400–450 nm)—should be systematically investigated across various skin color types. HEV is increasingly recognized as a significant contributor to skin pigmentation [10,69,70,71] and is also responsible for inducing IPD/PPD [17,72,73]. Understanding how these wavelengths contribute to melanin photomodification will be vital for developing comprehensive interventions to safeguard skin health.

## 4. Materials and Methods

### 4.1. Skin Samples

Normal human skin samples were obtained after breast reduction surgery from healthy women (age 45.7 ± 0.4) with the patients’ written informed consent in accordance with the Helsinki Declaration and with Article L. 1243-4 of the French Public Health Code. Patients’ written informed consents were collected and kept by the surgeon. The samples were blind coded before their reception and only the age, gender, and anatomical site of the samples were specified. The hair color and eye color of the subjects were not available. The authors did not participate in the sample collection. Given its special nature, surgical residue is subject to specific legislation included in the French Code of Public Health (anonymity, gratuity, sanitary/safety rules). This legislation does not require prior authorization by an ethics committee for sampling or use of surgical waste.

### 4.2. Colorimetry

A Check spectrocolorimeter (Datacolor, Montreuil, France) was used to measure the L* and b* parameters of the L*a*b* color space (Commission Internationale de l’Eclairage, 1976; https://www.cie.co.at/cie/ (accessed on 9 January 2026)), using the D65, 10°, SCI, d/8° specifications. L* is the luminance and b* is the yellow–blue component. The individual typology angle (ITA) was determined according to the formula ITA = (Arctan ((L* − 50)/b*)) × 180/π than allows the skin samples to be classified into one of six groups: Very light > 55° > Light > 41° > Intermediate > 28° > Tan > 10° > Brown > −30° > Dark [74,75]. Melanocompetent skin specimens with ITA values comprised between 22.17 and −52.05 (mean age 46) were included in the study as follows: Tan (*n* = 2), Brown (*n* = 11) and Dark (*n* = 5). For light skin types, Very light (*n* = 3) and Light (*n* = 12) specimens (mean age 44) were included based on their ITA value (between 41.7 and 57.2).

### 4.3. UVA1 Exposure

Samples obtained between 2 and 4 h after surgery were exposed to 60, 90, and 120 J/cm^2^ UVA1 (340–400 nm) using a 1000-watt xenon arc solar simulator (Oriel, Palaiseau, France) equipped with a dichroic mirror and a WG360 filter (Schott, Clichy, France). The UVA1 spectrum is illustrated in Figure S4. During transfer from the operating room, the skin was kept at 4 °C on gauze soaked with Phosphate-Buffered Saline (PBS, Waltham, MA, USA). During the UVA1 exposure and throughout the rest of the procedure, it was maintained at room temperature on PBS-soaked gauze. Eight-millimeter diameter punch biopsies were collected before, immediately after (designated as 0 h), and 2 h after exposure. For dark skin, six independent studies were performed in triplicate per condition (except for the 2 h post-120 J/cm^2^ exposure point, where *n* = 5) and results were averaged. For light skin, five independent studies were performed per dose.

### 4.4. Melanin Analysis by Image Analysis on Fontana–Masson-Stained Skin Sections

Punch biopsies were fixed and embedded in paraffin. Skin sections were stained with Fontana–Masson for histological visualization of melanin (dark and brown dots) after nuclear fast red counterstaining. Skin sections were captured cartographically with a NanoZoomer scanner (Hamamatsu, Palaiseau, France) at 20-fold magnification. An in-house image analysis software was used to obtain melanin index (MI) as the ratio of the surface covered by melanin over the epidermal surface including the stratum corneum.

### 4.5. Chemical Analysis of Melanins

Punch biopsies were incubated in 2 M NaBr (Sigma-Aldrich, St. Louis, MO, USA) at room temperature for 1 h, and the epidermis was separated from the dermis and lyophilized. Dried epidermal samples were homogenized in 700 µL water at a concentration of 6–10 mg/mL with Ten-Broeck homogenizer (Corning, Osaka, Japan). Aliquots of 100 μL water suspension (0.6–1 mg sample) were subjected to AHPO to analyze PTCA, PDCA, TTCA, and PTeCA [29,33,76], and HI hydrolysis to analyze 4-AHP and 3-AHP [30]. Free PTCA was analyzed as described in Ito et al. [29]. Aliquots of 200 μL water suspension were subjected to Soluene-350 solubilization to estimate the total melanin content based upon A500 absorbance [31]. A background of 0.003/mg skin due to proteins was subtracted from A500 values.

### 4.6. Statistical Analyses

Student’s *t*-tests were performed using Microsoft Excel 2025 version 16.104.1 (26010228) (Japan Microsoft Co., Tokyo, Japan). A *p* value of <0.05 was considered statistically significant.

## 5. Conclusions

In conclusion, this study provides the first comprehensive characterization of UVA1-induced structural modifications of melanins in human skin associated with IPD and PPD in both dark and light skin. Our study has two primary limitations: (i) ethical constraints precluding in vivo experimentation on human volunteers, necessitating the use of ex vivo skin, and (ii) that while the applied UVA doses were physiological and achievable, the 120 J/cm^2^ UVA dose was notably high.

We demonstrated that UVA1 triggers a complex interplay of photooxidation, photodegradation, and cross-linking within the melanin polymer. While lower exposure levels primarily induce photooxidative changes, higher doses lead to the progressive degradation of both eumelanin and pheomelanin markers. These chemical modifications occur in the absence of visible cytotoxicity, suggesting that UVA1 alters the skin melanin pigment before cellular damage becomes apparent.

Our findings reveal a dose-dependent susceptibility among melanin types, with eumelanin, and specifically its DHICA subunits, showing greater vulnerability to UVA1-induced impairment compared to DHI units and pheomelanin.

Taken together, by clarifying how UVA1 compromises the structural integrity of preexisting melanin, this research highlights the necessity for advanced photoprotection strategies that specifically mitigate melanin photomodification. Such strategies are essential to safeguard skin health against subsequent solar exposure, particularly given that the protective neo-melanogenesis associated with delayed tanning takes several days to occur.

## Data Availability

Data are contained within the article and Supplemental Materials.

## Supplementary Materials

The following supporting information can be downloaded at: https://www.mdpi.com/article/10.3390/ijms27093973/s1.

## Abbreviations

The following abbreviations are used in this manuscript:

UVA	Ultraviolet-A
IPD	Immediate pigment darkening
PPD	Persistent pigment darkening
EM	Eumelanin
PM	Pheomelanin
DHI	5,6-Dihydroxyindole
DHICA	5,6-Dihydroxyindole-2-carboxylic acid
DPPH	2,2-Diphenyl-1-picrylhydrazyl
AHPO	Alkaline hydrogen peroxide oxidation
HPLC	High-performance liquid chromatography
HI	Hydroiodic acid
PTCA	Pyrrole-2,3,5-tricarboxylic acid
PDCA	Pyrrole-2,3-dicarboxylic acid
PTeCA	Pyrrole-2,3,4,5-tetracarboxylic acid
SEM	Standard error of the mean
TTCA	Thiazole-2,4,5-tricarboxylic acid
4-AHP	4-Amino-3-hydroxyphenylalanine
3-AHP	3-Amino-4-hydroxyphenylalanine
A500	Absorbance at 500 nm
ITA	Individual topology angle
HES	Hematoxylin-Eosin-Saffron
dCPD	delayed cyclobutane pyrimidine dimer
HEV	High-energy visible light
ROS	Reactive oxygen species
PIH	Post-inflammatory hyperpigmentation
