# Supplementary material for: Unraveling UVA1-Induced Photomodifications of Eumelanin and Pheomelanin in Human Skin: Insights into Pigment Darkening
Source: Int J Mol Sci. 2026 Apr 29;27(9):3973. doi: 10.3390/ijms27093973 (PMC13163989; doi:10.3390/ijms27093973)
Supplement: Supplementary file 1 [file ijms-27-03973-s001.zip › ijms-4239104-supplementary.pdf]

Supplemental information

Unraveling UVA1-Induced Photo Modifications of Eumelanin and Pheomelanin in Human Skin: Insights into Pigment Darkening

Shosuke Ito, Juliette Sok, Yukiko Nakanishi, Kazumasa Wakamatsu and Sandra Del Bino

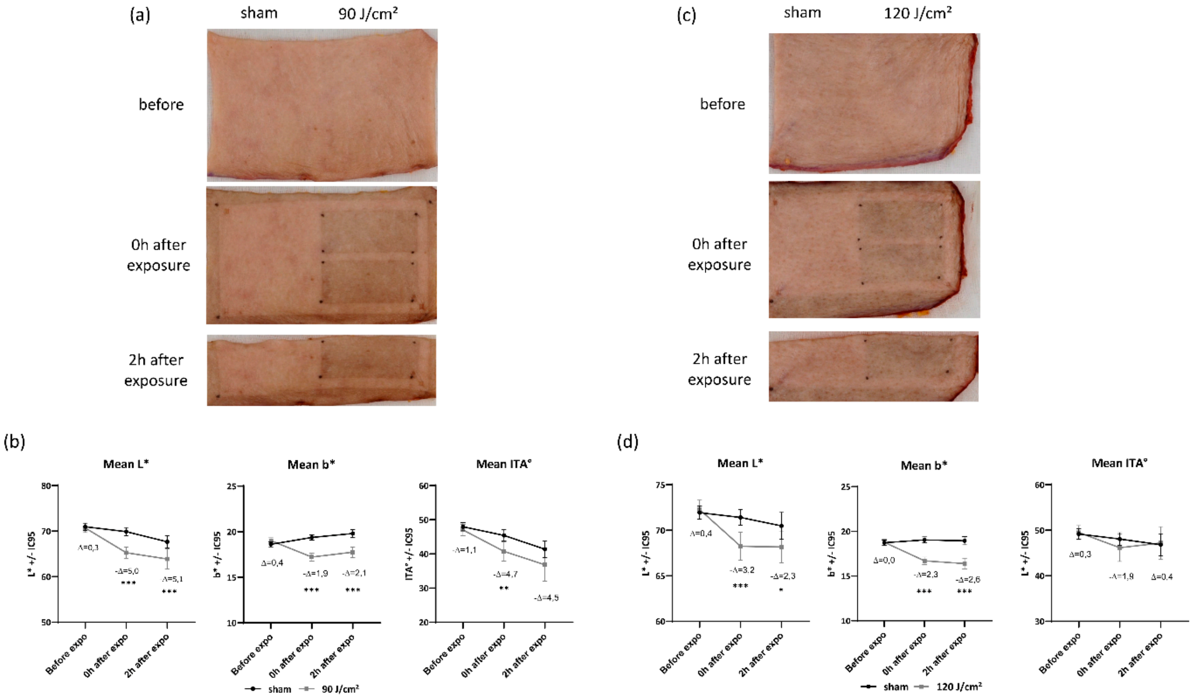

Figure S1. Macroscopic aspect of light *ex vivo* skin and colorimetric parameters after exposure to 90, and 120 J/cm² UVA1. (a, c) Photographs of *ex vivo* skin at sham or exposed site, before, immediately, 2 h after exposure at 90, and 120 J/cm² UVA1. (b, d) Changes in L*, b* parameters and Individual Typology Angle (ITA) before, immediately, 2 h after exposure to 90, and 120 J/cm² UVA1. Data were averaged from five independent experiments with standard error of the mean (SEM). Student's test for paired samples (two-tailed), * = $p < 0.05$, ** = $p < 0.01$, *** = $p < 0.001$.

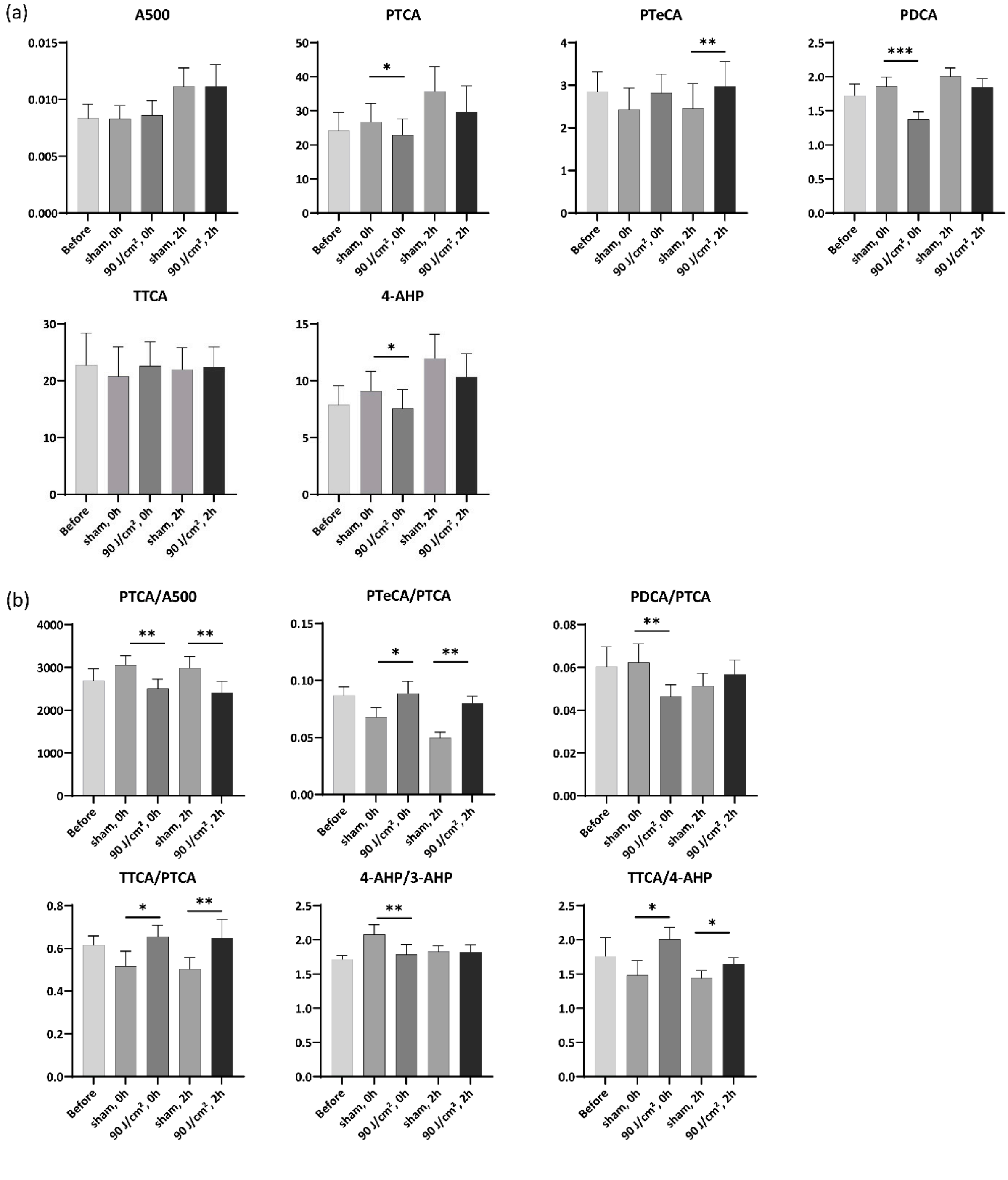

Figure S2. Melanin markers and ratios after exposure of light skin to 90 J/cm² UVA1. (a) Changes in melanin markers before, immediately, 2 h after exposure. (b) Changes in marker ratios before, immediately, 2 h after exposure. Data were averaged from six independent experiments with SEM. Student's test for paired samples (one-tailed), * = $p < 0.05$, ** = $p < 0.01$, *** = $p < 0.001$.

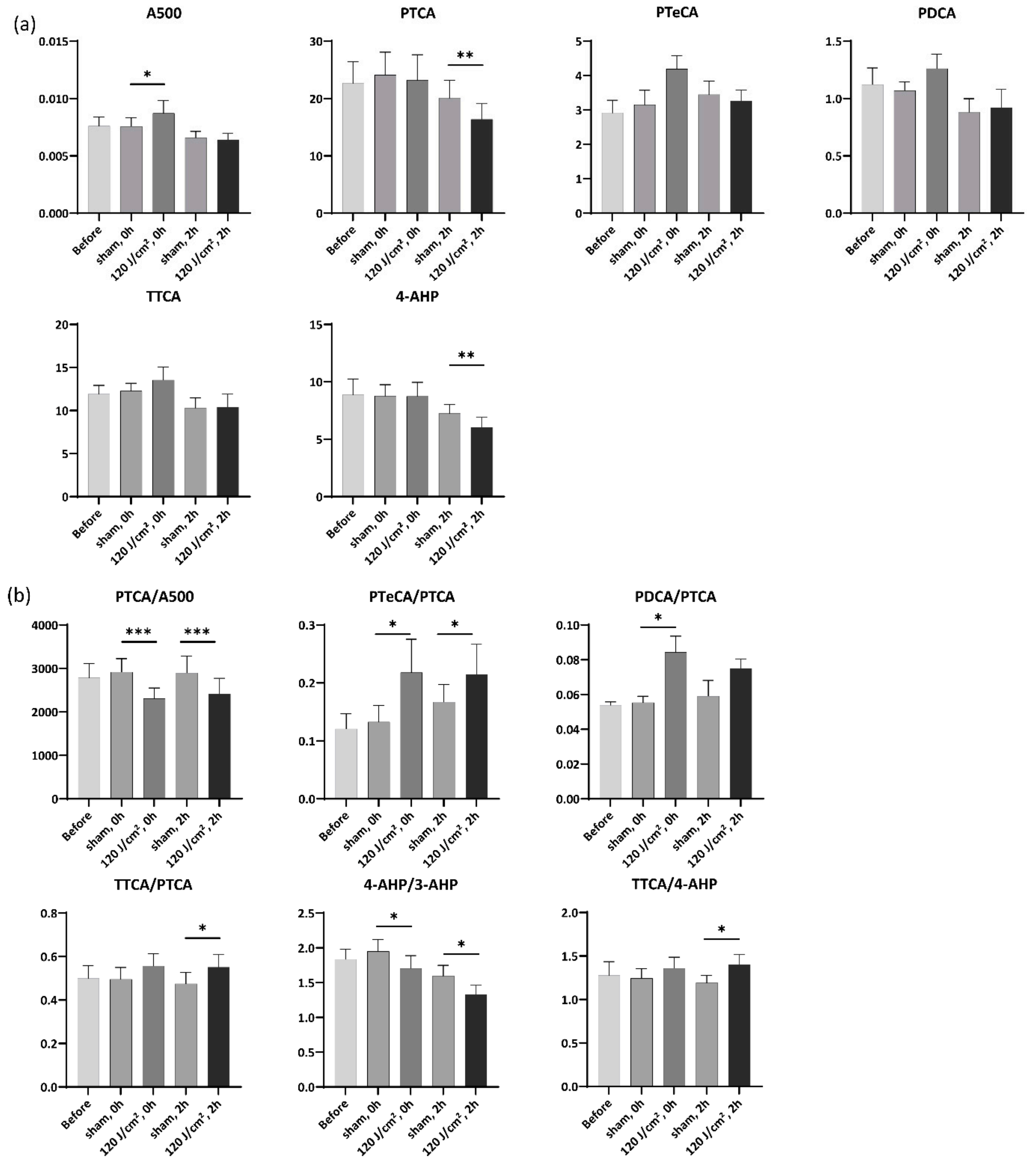

Figure S3. Melanin markers and ratios after exposure of light skin to 120 J/cm² UVA1. (a) Changes in melanin markers before, immediately, 2 h after exposure. (b) Changes in marker ratios before, immediately, 2 h after exposure. Data were averaged from six independent experiments with SEM. Student's test for paired samples (one-tailed), * = $p < 0.05$, ** = $p < 0.01$, *** = $p < 0.001$.

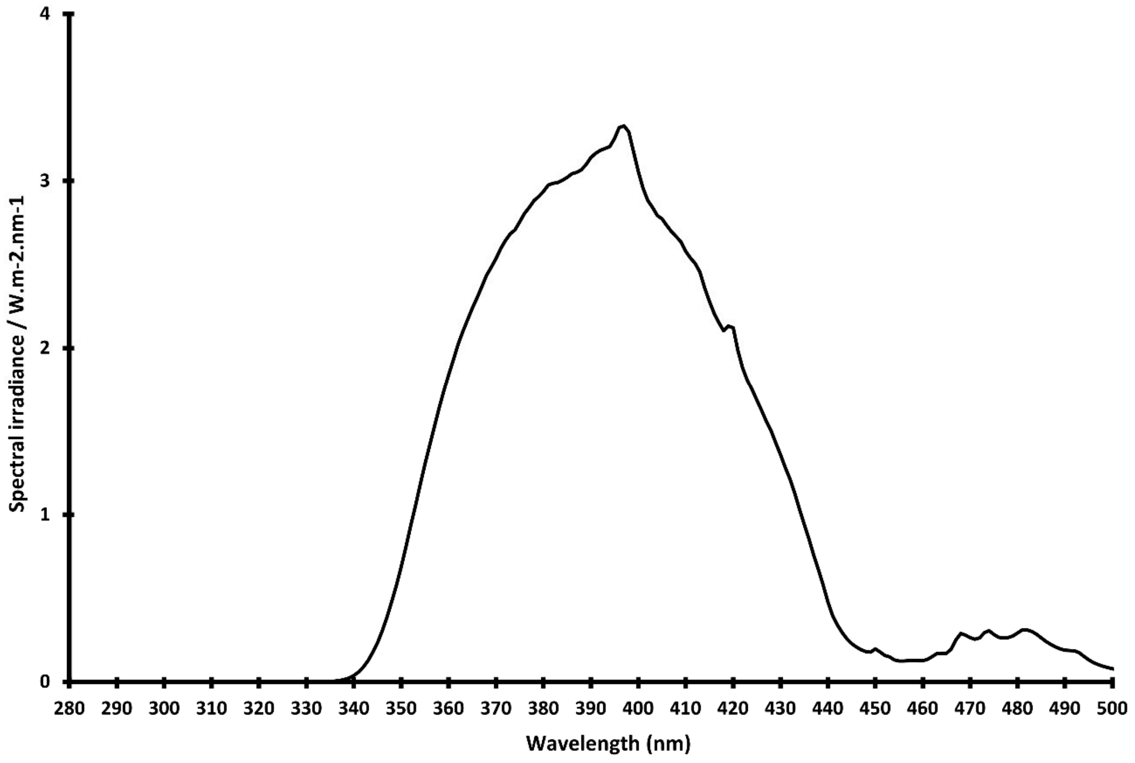

Figure S4. UVA1 spectrum delivered using a 1600 W Xenon lamp equipped with a dichroic mirror (Oriel, les Ulis, France) and a WG360 2mm thick filter (Schott, Clichy, France) cutting out wavelengths shorter than 340 nm. To deliver all UVA1 wavelengths, a part of the visible light spectrum (400-450 nm) could not be separated from the applied UVA1 spectrum. The spectral irradiance was measured using a spectroradiometer (Macam Photometrics, Livingston, UK).

Conditions (60 J/cm²)	A500	A650	PTCA	PDCA	TTCA	Free PTCA	PTeCA	4-AHP	3-AHP	A650 /A500	PTCA /A500	Free/Total PTCA	PTeCA /PTCA	4-AHP /3-AHP	TTCA /4-AHP
Before exposure, 0 J/cm²	0.0470 (0.0027)	0.0112 (0.0007)	141.5 (7.74)	5.15 (0.464)	44.8 (2.90)	7.54 (0.76)	11.7 (0.84)	28.1 (3.21)	11.4 (1.00)	0.237 (0.0024)	3024 (92.1)	0.0649 (0.0155)	0.0860 (0.0073)	2.39 (0.130)	2.03 (0.265)
0h after exposure, 0 J/cm²	0.0444 (0.0020)	0.0105 (0.0006)	133.2 (7.09)	5.05 (0.391)	43.2 (2.29)	8.46 (1.12)	10.9 (0.59)	26.7 (2.85)	10.9 (0.89)	0.235 (0.0040)	3000 (88.9)	0.0755 (0.0224)	0.0852 (0.0060)	2.40 (0.114)	2.04 (0.255)
0h after exposure, 60 J/cm²	0.0466 (0.0016)	0.0113 (0.0005)	124.5 (4.48)	4.69 (0.377)	45.1 (2.15)	9.14 (1.79)	12.4 (0.76)	27.4 (3.08)	11.3 (1.03)	0.241 (0.0043)	2683 (63.3)	0.0801 (0.0220)	0.1015 (0.0081)	2.38 (0.104)	2.19 (0.319)
2h after exposure, 0 J/cm²	0.0428 (0.0021)	0.0103 (0.0006)	126.6 (7.30)	4.69 (0.324)	42.9 (1.39)	7.69 (1.34)	11.3 (0.62)	27.2 (3.18)	11.4 (1.02)	0.241 (0.0050)	2943 (76.9)	0.0673 (0.0122)	0.0931 (0.0067)	2.33 (0.108)	2.06 (0.247)
2h after exposure, 60 J/cm²	0.0481 (0.0029)	0.0119 (0.0009)	128.4 (6.78)	5.04 (0.387)	47.1 (2.59)	7.88 (0.70)	12.4 (0.63)	28.0 (3.58)	11.4 (1.18)	0.242 (0.0053)	2694 (64.0)	0.0690 (0.0120)	0.0994 (0.0056)	2.37 (0.104)	2.28 (0.322)
**Conditions (90 J/cm²)**	**A500**	**A650**	**PTCA**	**PDCA**	**TTCA**	**Free PTCA**	**PTeCA**	**4-AHP**	**3-AHP**	**A650 /A500**	**PTCA /A500**	**Free/Total PTCA**	**PTeCA /PTCA**	**4-AHP /3-AHP**	**TTCA /4-AHP**
Before exposure, 0 J/cm²	0.0594 (0.0051)	0.0141 (0.0013)	183.7 (16.6)	5.84 (0.482)	54.0 (4.25)	6.82 (0.81)	15.3 (1.24)	28.3 (2.42)	12.0 (0.99)	0.235 (0.0036)	3095 (74.3)	0.0446 (0.0055)	0.0853 (0.0046)	2.37 (0.075)	2.20 (0.229)
0h after exposure, 0 J/cm²	0.0534 (0.0034)	0.0128 (0.0009)	164.5 (10.4)	5.30 (0.415)	51.3 (2.78)	7.02 (1.03)	13.5 (1.01)	27.2 (2.78)	11.4 (1.03)	0.238 (0.0029)	3093 (69.8)	0.0456 (0.0044)	0.0821 (0.0041)	2.33 (0.071)	2.30 (0.275)
0h after exposure, 90 J/cm²	0.0553 (0.0037)	0.0130 (0.0009)	153.0 (13.6)	4.71 (0.359)	55.8 (4.37)	6.97 (1.08)	14.1 (1.13)	26.3 (2.02)	10.7 (0.66)	0.233 (0.0027)	2725 (69.6)	0.0524 (0.0041)	0.0939 (0.0049)	2.43 (0.056)	2.39 (0.250)
2h after exposure, 0 J/cm²	0.0552 (0.0032)	0.0130 (0.00087)	168.8 (8.73)	5.41 (0.294)	55.1 (3.17)	6.98 (0.84)	14.3 (0.87)	28.1 (2.68)	11.4 (0.98)	0.236 (0.0032)	3099 (89.9)	0.0443 (0.0031)	0.0852 (0.0035)	2.43 (0.057)	2.40 (0.303)
2h after exposure, 90 J/cm²	0.0492 (0.0033)	0.0116 (0.0008)	136.6 (12.9)	4.36 (0.337)	52.6 (4.23)	7.29 (1.13)	13.3 (0.99)	22.0 (1.73)	9.14 (0.59)	0.235 (0.0028)	2724 (67.5)	0.0613 (0.0074)	0.1002 (0.0044)	2.37 (0.057)	2.68 (0.283)
**Conditions (120 J/cm²)**	**A500**	**A650**	**PTCA**	**PDCA**	**TTCA**	**Free PTCA**	**PTeCA**	**4-AHP**	**3-AHP**	**A650 /A500**	**PTCA /A500**	**Free/Total PTCA**	**PTeCA /PTCA**	**4-AHP /3-AHP**	**TTCA /4-AHP**
Before exposure, 0 J/cm²	0.0602 (0.0049)	0.0143 (0.0013)	182.6 (17.3)	5.08 (0.435)	60.1 (4.87)	4.92 (1.29)	13.9 (1.28)	30.9 (3.00)	13.0 (1.15)	0.235 (0.0030)	2990 (69.9)	0.0296 (0.0094)	0.0771 (0.0026)	2.36 (0.055)	2.08 (0.182)
0h after exposure, 0 J/cm²	0.0550 (0.0046)	0.0133 (0.0011)	160.0 (16.0)	4.54 (0.378)	52.2 (4.52)	4.05 (1.42)	13.1 (1.12)	28.0 (2.34)	11.7 (0.92)	0.244 (0.0030)	2866 (65.8)	0.0269 (0.0131)	0.0773 (0.0024)	2.39 (0.050)	1.92 (0.158)
0h after exposure, 120 J/cm²	0.0546 (0.0052)	0.0130 (0.0013)	133.5 (15.8)	4.29 (0.352)	52.0 (4.92)	4.15 (1.40)	11.9 (1.24)	26.0 (2.49)	10.7 (0.99)	0.236 (0.0027)	2361 (77.6)	0.0368 (0.0197)	0.0927 (0.0038)	2.42 (0.043)	2.05 (0.178)
2h after exposure, 0 J/cm²	0.0580 (0.0048)	0.0139 (0.0013)	174.6 (16.3)	4.80 (0.399)	59.8 (4.71)	5.04 (1.38)	13.4 (1.10)	29.7 (2.91)	12.0 (1.11)	0.238 (0.0030)	2996 (72.2)	0.0317 (0.0110)	0.0789 (0.0023)	2.47 (0.062)	2.11 (0.163)
2h after exposure, 120 J/cm²	0.0527 (0.0047)	0.0128 (0.0012)	120.4 (15.4)	3.86 (0.388)	51.1 (4.12)	5.01 (1.35)	11.8 (1.23)	23.0 (1.85)	9.64 (0.81)	0.242 (0.0035)	2315 (84.6)	0.0456 (0.0156)	0.0975 (0.0049)	2.41 (0.061)	2.31 (0.175)

Table S1. Summary of the results of UVA1 exposure on *ex vivo* dark skin. Average (and SEM) values for marker level and their ratios. Values are average (and SEM) for six independent experiments on dark skin. Units of marker are ng/mg sample (for A500 and A650, absorbance/mg sample).

Dark skin	A500		PTCA		PTeCA		Free PTCA		PTCA/A500		PTeCA/PTCA		Free/Total PTCA		PDCA		PDCA/PTCA		TTCA/PTCA		TTCA		4-AHP		4/3 AHP		TTCA/4-AHP	
Doses	0h	2h	0h	2h	0h	2h	0h	2h	0h	2h	0h	2h	0h	2h	0h	2h	0h	2h	0h	2h	0h	2h	0h	2h	0h	2h	0h	2h
60 J/cm²	1.05	1.12	0.93	1.01	**1.13**	1.10	1.02	1.07	**0.89**	**0.92**	**1.19**	1.07	1.00	0.99	0.93	1.07	1.03	1.08	**1.09**	1.06	1.04	1.10	1.02	1.03	0.99	1.02	1.08	1.11
90 J/cm²	1.04	0.89	**0.93**	**0.81**	1.04	0.93	0.99	0.95	**0.88**	**0.88**	**1.14**	**1.18**	1.04	**1.19**	**0.89**	**0.81**	0.99	1.02	**1.17**	**1.20**	1.09	0.95	0.97	**0.78**	**1.04**	0.98	1.04	**1.12**
120 J/cm²	0.99	0.91	**0.83**	**0.73**	0.98	0.88	1.00	1.02	**0.82**	**0.77**	**1.20**	**1.23**	**1.23**	**1.32**	0.94	**0.80**	**1.19**	**1.15**	**1.22**	**1.24**	0.99	**0.86**	**0.93**	**0.77**	1.01	0.98	1.07	**1.10**

Light Skin	A500		PTCA		PTeCA		Free PTCA		PTCA/A500		PTeCA/PTCA		Free/Total PTCA		PDCA		PDCA/PTCA		TTCA/PTCA		TTCA		4-AHP		4/3 AHP		TTCA/4-AHP	
Doses	0h	2h	0h	2h	0h	2h	0h	2h	0h	2h	0h	2h	0h	2h	0h	2h	0h	2h	0h	2h	0h	2h	0h	2h	0h	2h	0h	2h
60 J/cm²	0.90	**1.28**	**0.83**	0.90	0.90	**1.17**	ND	ND	**0.92**	0.74	**1.17**	**1.29**	ND	ND	**0.75**	0.97	**0.91**	1.338	**1.13**	**1.04**	0.94	**1.06**	**0.81**	0.90	0.93	0.97	**1.21**	**1.17**
90 J/cm²	1.04	1.00	**0.86**	0.83	1.16	**1.21**	ND	ND	**0.82**	**0.81**	**1.30**	**1.61**	ND	ND	**0.74**	0.92	**0.74**	1.11	**1.27**	**1.28**	1.09	1.02	**0.83**	0.86	**0.86**	0.99	**1.36**	**1.14**
120 J/cm²	**1.15**	0.96	0.96	**0.81**	1.33	0.94	ND	ND	**0.79**	**0.83**	**1.64**	**1.28**	ND	ND	1.18	1.05	**1.50**	1.27	1.12	**1.16**	1.10	1.01	1.00	**0.83**	**0.88**	**0.83**	1.10	**1.17**

Table S2. Melan markers and ratios. Average data for dark and light skin exposed to 60, 90 or 120 J/cm² UVA1. Values are fold change (FC), defined as the ratio of marker levels in UVA1-exposed samples relative to sham-treated controls. FC are highlighted in green if FC > 1.00 or in red if FC < 1.00 and significant FC are bold. ND corresponds to not detectable.
